# Evaluation of the Effects of Folic Acid Combined with Atorvastatin on the Poststroke Cognitive Impairment by Low-Rank Matrix Denoising Algorithm-Based MRI Imaging

**DOI:** 10.1155/2022/9540701

**Published:** 2022-03-04

**Authors:** Yancui Li, Zhou Fang, Jianghua Li, Jing Wang, Xi Wang, Xiang Li, Fang Fang

**Affiliations:** ^1^Department of Pharmaceutics, The First Affiliated Hospital of Jiamusi University, Jiamusi 154002, Heilongjiang, China; ^2^Department of Otolaryngology, The First Affiliated Hospital of Jiamusi University, Jiamusi 154002, Heilongjiang, China; ^3^Department of Pharmaceutics, Hongda Hospital of Jiamusi University, Jiamusi 154002, Heilongjiang, China

## Abstract

This research aimed to study the optimization effects of the low-rank matrix denoising (LRMD) algorithm based on the Gaussian mixture model (GMM) on MRI images of stroke patients, aiming to evaluate the effects of atorvastatin combined with folic acid on poststroke cognitive impairment (PSCI) in patients with ischemic stroke. First, the GMM-based low-rank matrix denoising (LRMD) algorithm was constructed and applied to process MRI images of 64 patients with ischemic stroke. Then, the MRI images before and after processing were compared for the denoising degree and quality. An image with 5% noise was not as clear as an MRI image with 1% noise, and the effects of atorvastatin combined with folic acid on PSCI in patients with ischemic stroke were discussed. It was found that the denoising degree of MRI images processed by the GMM-based LRMD algorithm was significantly improved, the image quality was significantly enhanced (*P* < 0.05), and the diagnosis accuracy and efficiency of stroke patients were heightened. Atorvastatin combined with folic acid reduce the homocysteine (HCY) and total cholesterol (TC) levels, as well as Montreal Cognitive Scale (MOCA) scores of PSCI patients (*P* < 0.05). In conclusion, the MRI images processed by the LRMD algorithm have good quality. Folic acid combined with atorvastatin can effectively reduce HCY and TC levels, thereby alleviating PSCI of stroke patients.

## 1. Introduction

Stroke is a cerebrovascular disease, arising from the organic changes of the cerebral vessels, such as blockages and malformations. Its attack is sudden, which can lead to poststroke cognitive impairment [1]. Clinically, stroke usually falls into two types: ischemic stroke and hemorrhagic stroke. The former will cause muscle stiffness, weakness of the limbs, and damage to cognitive functions, such as memory and thinking, bringing great inconvenience to patients' daily lives [2]. PSCI is a kind of behavioral cognitive impairment caused by stroke, and it has attracted the attention of many researchers [3]. Studies have shown that the emergence of PSCI is related to the lack of nutrition in patients, and appropriate supplementation of nutrients can effectively prevent cognitive system dysfunction [4]. The research results of Ma et al. [5] showed that the lack of nutrients such as folic acid can accelerate the cognitive decline of patients with ischemic stroke. Enderami et al. [6] evaluated the folate levels of patients with cognitive impairment and found that patients with low folate levels are more likely to have memory problems, and either folic acid or in combination with other drugs can improve the body's cognitive level.

Nowadays, imaging technologies such as computed tomography (CT) and magnetic resonance imaging (MRI) are widely used in medical image analysis. As a result, the detection rate of malignant tumors has also generally increased, and the survival period of patients with malignant tumors begins to increase [7]. Among them, MRI imaging technology is a common method to diagnose brain tumors. Various imaging parameters produce various different modal maps, providing rich information for the diagnosis and treatment of brain tumors [8]. MRI is currently the only nonradiation and noninvasive imaging technology for cerebrovascular diseases, and it is sensitive in the differential diagnosis of intracranial aneurysms. It can show the vascular structure without using a contrast agent. Studies have pointed out that the traditional MRI imaging and transmission processes will be interfered by Rician noise, and effective denoising processing can improve the quality of MRI images, thereby improving the diagnostic efficiency [9].

There have been many reports on denoising algorithms for MRI images, including filtering methods, image block prior denoising algorithms, and low-rank matrix factorization (LRMF) based on image sparsity [10]. Xie et al. [11] found that the Gaussian mixture model (GMM) can easily construct prior noise-free images. Low-rank matrix factorization (LRMF) aims to find specific data items of a matrix that are infinitely close to a noisy image. In this study, a Gaussian mixture model (GMM)-based low-rank matrix denoising (LRMD) algorithm was used to process the MRI images of stroke patients, and its optimization effects were evaluated. Then, the effects of atorvastatin combined with folic acid on the PSCI of patients with ischemic stroke were explored, in order to further understand the role of folic acid combined with atorvastatin in the PSCI and treatment of stroke patients.

## 2. Materials and Methods

### 2.1. Research Subjects

Sixty-four patients with ischemic stroke from April 2019 to June 2020 were selected as research subjects, including 32 males and 48 females. All of them had the MRI examination. They were aged between 22 and 81 years old, with an average age of 52 years. After the patients were admitted to the hospital, their cognitive function was evaluated using Montreal Cognitive Assessment (MOCA) scale. All subjects were followed up at 3 and 6 months after the onset of ischemic stroke using the MOCA scale. This study has been approved by the ethics committee of hospital, and the patients and their families signed the informed consent form.

The inclusion criteria were as follows: (I) patients aged ≥18 years; (II) patients diagnosed with acute ischemic stroke as per the diagnostic criteria in *Neurology* (7th Edition, People's Medical Publishing House) [12]; (III) the time from first onset to admission was ≤7 days; (IV) those who can cooperate with medical staff to perform scale scoring and MRI examinations; (V) basic information was complete; and (VI) the patient was willing to cooperate in the follow-up.

The exclusion criteria were as follows: (I) cognitive function decline caused by previous nonvascular factors; (II) those accompanied by mental illnesses, such as depression and schizophrenia; (III) those accompanied by severe limb movement, hearing, and language impairment and unable to cooperate with the medical staff in the scale assessment; (IV) those who cannot complete head magnetic resonance examination due to the presence of metal foreign bodies such as steel plates, stents, cardiac pacemakers, and claustrophobia; (V) those suffering from malignant tumors, acute myocardial infarction, and other serious diseases of the system; and (VI) those who refused to participate in the registration investigation.

### 2.2. The Grouping Methods

The patients were divided into the non-PSCI group (group A) and the PSCI group according to MOCA results. According to whether to give atorvastatin combined with folic acid treatment, they were randomly divided into two groups: the PSCI untreated group (group B) and the PSCI treated group (group C). In group A, there were 20 subjects, including 14 males and 6 females, aged 66 years old. In group B, there were 22 cases, including 12 males and 10 females, aged 67 years. In group C, there were 22 patients, including 11 males and 11 females, aged 67 years old. Group A received placebo treatment; group B received placebo treatment; and group C took 15 mg of folic acid combined with 20 mg of atorvastatin orally, once a night. The drug intervention cycle lasted for 6 months. Then, the MOCA score, plasma and serum levels were detected.

### 2.3. Imaging Examination

After the patient was admitted to the hospital, the unenhanced MRI scan and susceptibility imaging were performed within 3 days. Cerebral microbleeds (CMBs) are an imaging manifestation of cerebral small vessel disease, showing circular uniform low signals with a diameter of 2∼5 mm and clear borders [13]. This is due to the escape of red blood cells from the blood vessel and then being swallowed by macrophages to form hemosiderin [14]. Two imaging physicians who were blinded read the films to find the location of the cerebral infarction and whether there were CMBs. Any inconsistencies were resolved by inviting a senior medical imaging physician to arbitrate.

### 2.4. GMM-Based Clustering of MRI Images of Stroke Patients

The MRI images of different organs are similar, and it is difficult to distinguish image blocks because of the similar interference signals of different structures in intracranial MRI images of stroke patients. Therefore, the GMM-based clustering method is used to cluster the MRI images of intracranial cerebral hemangioma together with the prior method of noise-free MRI image blocks. First, the MRI image is set as *S* and then segmented based on structural similarity. Finally, the segmentation results are merged (*n* image blocks) into a set, that is, PS=(*P*_1_*S*， …, *P*_*i*_*S*,…, *P*_*n*_*S*). In this set, *P*_*i*_*S* represents the matrix corresponding to the first image block. If the image block *PS* can be divided into *w* categories, the probability of the *P*_*i*_*S* image block can be expressed by GMM, and the equation is as follows:

(1) $$\begin{array}{c} p\left(P_{i}S|\Phi \right)=\overset{H}{\underset{h=1}{\sum }}w_{h}p_{h}\left(P_{i}S|\mu_{h,}\sum^{}h\right), \end{array}$$

where *H* represents the GMM containing *H* Gaussian classes, *h*  represents the number of corresponding Gaussian classes, *w* represents the weight, *μ* represents the mean, Σ is the reference covariance matrix, and Φ represents the set composition of the mean. *μ*, covariance matrix Σ, and the weight *w* of each Gaussian class satisfy the following equation. In addition, the probability density function of the h^th^ Gaussian class is used, expressed as follows:

(2) $$\begin{array}{c} p\left(P_{i}S|\mu_{h,}\sum^{}h\right)=c\cdot \;\exp\left(\left(-\frac{1}{2}{\left(P_{i}S-\mu_{h}\right)}^{T}\overset{-1}{\underset{h}{\sum }}\left(P_{i}S-\mu_{h}\right)\right.\right), \end{array}$$

where *c* represents the normalization constant, and the negative exponent represents the correlation between *P*_*i*_*S*  and *μ*_*h*,_.On this basis, *D*=(*d*_1_, *d*_2_,…, *d*_*h*_), *d*_*i*_ ∈ {1,2,…, *R*} class labels are used to simplify expressions. *p*(*P*_*i*_*S*, *d*_*i*_=*h|*Φ) indicates that *P*_*i*_*S*(*i*,…, *h*) is independent under the GMM parameter setting. In the GMM parameter set, the probability of PS clustering to class *R* is calculated as follows:

(3) $$\begin{array}{c} p\left(\text{PS},D|\Phi \right)=\overset{h}{\underset{i-1}{\prod }}p\left(P_{i}S,d_{i}|\Phi \right). \end{array}$$

**Figure 1:**
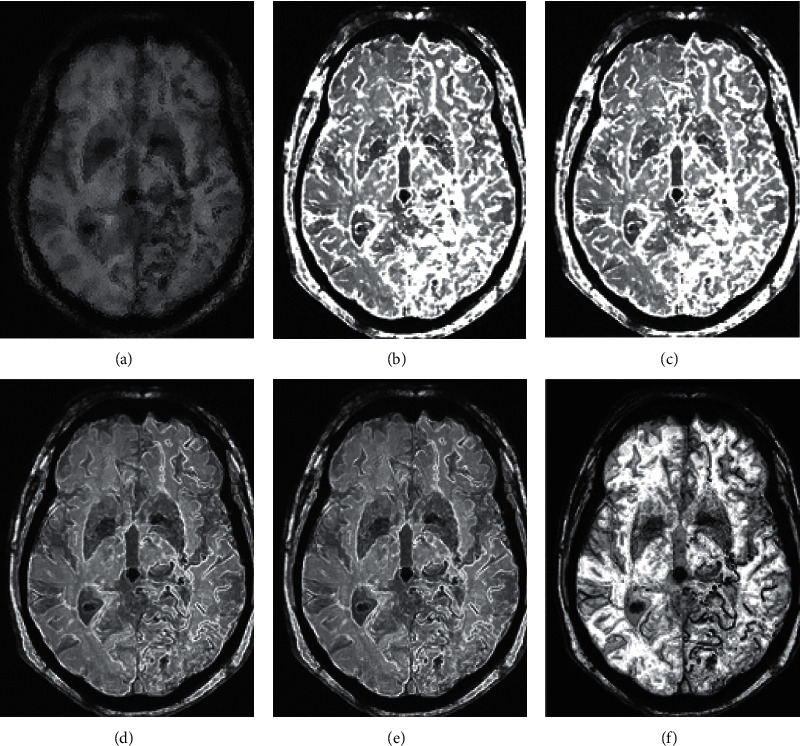
GMM-based clustering results of MRI images of stroke patients. *Note.* Figures (a), (b), (c), (d), (e), and (f) were images with 10% noise; clustering results of noisy images; clustering results generated by iterations; clustering results after 3 circles of iterations; clustering results after 6 circles of iterations and the denoised image.

The final clustering results of MRI images of cerebral aneurysms based on GMM are shown in Figure 1.

### 2.5. LRMF Denoising Algorithm Based on Image Block Prior

The MRI image of the brain aneurysm with given noise is divided into blocks, and then *P*=(*P*_1_*S*, *P*_*i*_*S*,…, *P*_*h*_*S*) is obtained. Assuming that the GMM parameter set is obtained by learning the information of noise-free MRI image blocks, *PS* is divided into *H* categories according to the prior knowledge of GMM. ${\overset{-}{P}}_{a}X=\left[P_{h_{1}},...,P_{h_{d\left(h\right)}}\right]$ refers to a matrix composed of all image blocks in the *H*^th^ category, and d(h) represents the number of all similar image blocks in the *H*^th^ category. The image blocks in the same Gaussian class contain similar structural information, so ${\overset{-}{P}}_{h}S$ can be decomposed into the following equations:

(4) $$\begin{array}{c} {\overset{-}{P}}_{h}S=Q_{h}+T_{h}, \end{array}$$

where *Q*_h_ represents a low-rank *matrix*, *T*_h_ represents a noise matrix, and a low-rank matrix represents image data after denoising. Assuming that the noise of each pixel in the image is independent and uniformly distributed, the energy function can be expressed as follows:

(5) $$\begin{array}{c} E\left(Q_{h}\right)=\tau Q_{h*}+\frac{1}{\sigma^{2}}{\overset{-}{P}}_{h}S-Q_{\text{hF}}^{2}, \end{array}$$

where *τ* represents the normal number, and *σ* represents the standard deviation of the noise; *Q*_*h*_ _*∗*_ is the matrix kernel norm, and ${\overset{-}{P}}_{h}S-Q_{h}{\;}_{F}^{2}$ is the norm of matrix Frobenius.The rank minimization in (5) can be solved by minimizing the weighted kernel norm. Hence, for a given noise MRI image, a noise-free MRI image can be reconstructed on the basis of a prior denoising model of the noise-free MRI image.

### 2.6. Evaluation Indexes of Stroke MRI Image Processed by the LRMD Algorithm

The quality of MRI images of cerebral aneurysms was evaluated by factoring into root mean square error (RMSE), peak signal-to-noise ratio (PSNR), and structural similarity (SSIM). MSE and PSNR describe the error between the target image *z* and the original image *x* from the perspective of pixel values, while SSIM takes into account the characteristics of the human visual system, making the evaluation results more in line with the human senses.

### 2.7. Cognitive Function Evaluation Indexes

The changes of plasma index homocysteine (HCY), serum index total cholesterol (TC), and MOCA scores before and after treatment were compared between untreated PSCI patients and non-PSCI patients.

The diagnostic criteria of PSCI were as follows: (I) PSCI that occurred within half a year after the occurrence of the stroke; (II) PSCI (MOCA total score <26 points) caused by the stroke.

For the determination of plasma HCY, 4 mL of early morning fasting blood was drawn from the cubital vein of the subject, and then transferred to a common desiccator, stored at 4°C. Then, it was centrifuged within 1 hour (3000 rpm, 10 min), and hemorrhagic cells and plasma were separated. Next, the 0.5 mL of plasma in the upper layer was transferred into a 1 mL centrifuge tube, and then stored at −20°C to facilitate the batch measurement of plasma HCY.

For determination of serum TC, 10 mL of fasting venous blood was drawn and transferred to an ordinary drying tube. After 15 minutes, the blood sample was centrifuged in a high-speed automatic balance for 10 minutes at 3000 rpm. Subsequently, the upper layer of plasma was taken and transferred into a test tube. An automatic biochemical analyzer was used to detect the level of serum TC.

### 2.8. Statistical Analysis

Statistical analysis was performed using SPSS 24.0 software, expressed as mean ± standard deviation (‾*x* ± *s*). The *χ*^2^ test method was used to process the data, with *α* = 0.05, and *P* < 0.05 was the threshold for significance.

## 3. Results

### 3.1. Changes in MRI Images of Stroke Patients before and after Processing by GMM-Based LRMD Algorithm

**Figure 2:**
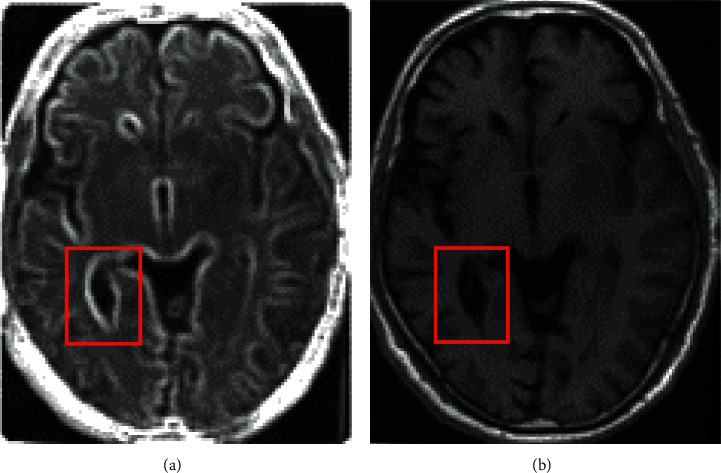
Comparison of MRI images before and after being processed by the GMM-based LRMD algorithm. (a) The image before denoising, and (b) the image after denoising. The red box indicates intracranial hemorrhage.

Figure 2 shows the MRI images of patients with ischemic stroke tumors before and after processing by the GMM-based LRMD algorithm. Compared with the MRI image before denoising, the texture of the MRI image after denoising was clearer.

**Figure 3:**
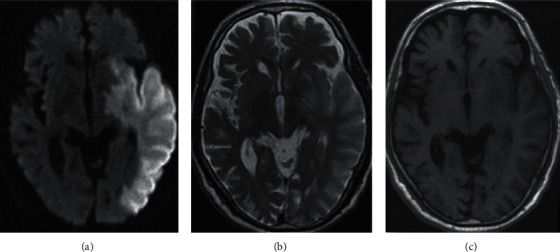
Comparison of MRI images of stroke patients under different noise intensities. (a) 5%, (b) 1%, and (c) noise-free MRI images.

Figure 3 shows the clarity of the MRI image of the same patient. The image with 5% noise was not as clear as the MRI image with 1% noise. Therefore, denoising processing can greatly change the visual clarity of the MRI image.

### 3.2. Comparison of MRI Image Quality Indexes of Stroke Patients under Different Noise Intensities

**Figure 4:**
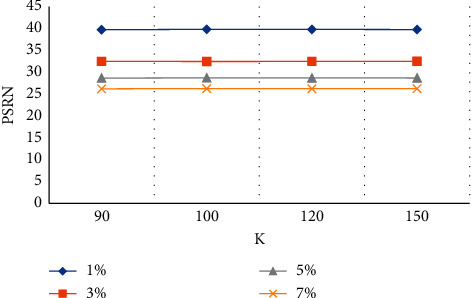
PSRN values at different *K* values.

The MRI image quality indexes of stroke patients showed that under the same noise intensity, the difference of the PSNR under different *K* values was very small, as shown in Figure 4.

**Figure 5:**
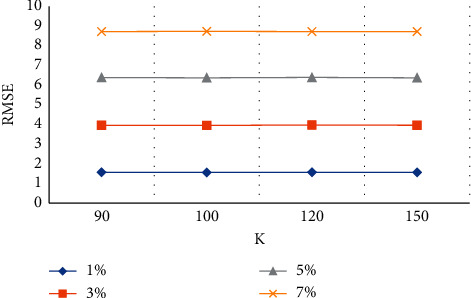
RMSE values at different *K* values.

Figure 5 shows that as the noise intensity increased from 1% to 7%, the RMSE increased from 1.57 dB to 8.72 dB, indicating that greater noise intensity led to lower similarity between the processed MRI image and the original image.

### 3.3. MRI Image Quality Processed by a GMM-Based LRMD Algorithm

**Figure 6:**
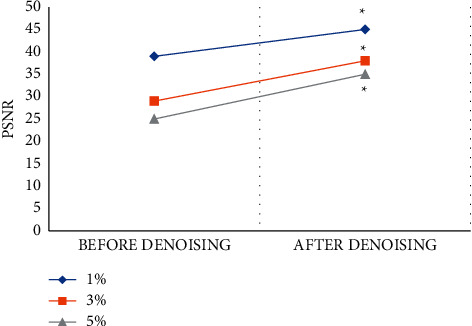
Comparison of PSNR scores of MRI images before and after denoising. Note:  ^*∗*^ indicates that there was a significant difference between the PSNR score after denoising and the PSNR score before denoising (*P* < 0.05).

The PSNR value of the MRI image processed by the GMM-based LRMD algorithm was compared with that of the original MRI image, as shown in Figure 6. The PSNR value of the MRI images processed by the GMM-LRMD algorithm increased by an average of 12.5 dB, indicating that the LRMD algorithm can significantly improve PSNR and that the difference was statistically significant (*P* < 0.05). If the denoised MRI image is closer to the original image, the image quality is higher and it is easier to diagnose an ischemic stroke.

**Figure 7:**
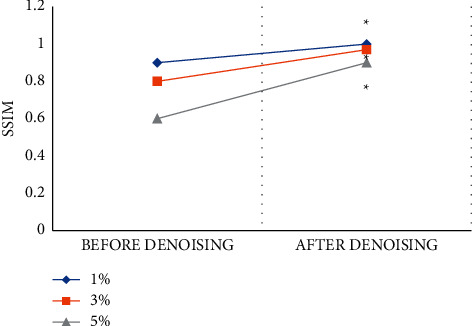
Comparison of SSIM scores of MRI images before and after denoising. Note:  ^*∗*^ indicates that there was a significant difference between the SSIM score after denoising and the SSIM score before denoising (*P* < 0.05).

Figure 7 compares SSIM values of MRI images before and after being processed by the LRMD algorithm. As the degree of noise increased, the SSIM value of the LRMD algorithm was reduced from 0.9 to 0.6 dB, while it was always less than 1. A lower noise degree indicates that the closer SSIM value after denosing is closer to 1. In other words, higher similarity between the original image and the target image indicates higher quality of the original image.

### 3.4. HCY Levels the Three Groups of Patients before and after Treatment

**Figure 8:**
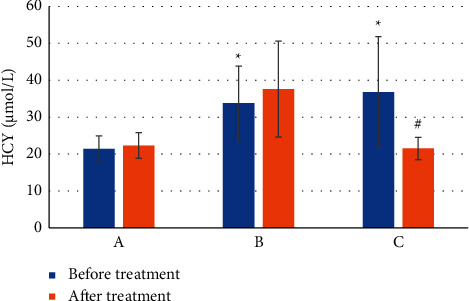
Comparison of the HCY levels of the three groups of patients before and after treatment. Note:  ^*∗*^ means there was a significant difference versus group A (*P* < 0.05). # indicates that there was a significant difference before and after treatment in the same group (*P* < 0.05).

Figure 8 shows the HCY values of the patients before and after treatment. Before treatment, the average HCY of patients in group A was 21.4 ± 3.52 *μ*mol/L, that of patients in group B was 33.8 ± 2.44 *μ*mol/L, and that in group C was 36.8 ± 3.05 *μ*mol/L. Obviously, the HCY levels of patients in groups A and B were significantly different (*P* < 0.05), the HCY levels of patients in groups A and C were also significantly different (*P* < 0.05), but the differences in HCY levels of patients in groups B and C were not significant (*P* > 0.05). After treatment, the average HCY of patients in group A was 22.3 ± 2.78 *μ*mol/L, that in group B was 37.6 ± 2.56 *μ*mol/L, and that in group C was 21.5 ± 3.17 *μ*mol/L. The HCY values of patients in groups A and B were not significantly different before and after treatment (*P* > 0.05), while the HCY values of patients in group C were significantly different before and after treatment (*P* < 0.05).

### 3.5. TC Levels of the Three Groups of Patients before and after Treatment

**Figure 9:**
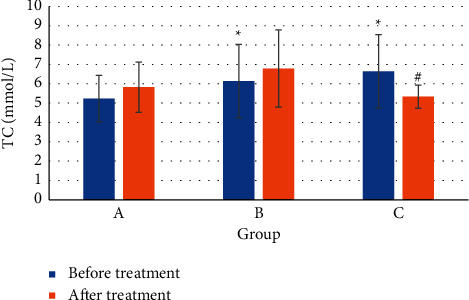
Comparison of the TC levels of the three groups of patients before and after treatment. Note:  ^*∗*^ means there was a significant difference versus group A (*P* < 0.05). # indicates that there was a significant difference before and after treatment in the same group (*P* < 0.05).

Figure 9 shows the serum TC values of the patients before and after the treatment. Before treatment, the average TC of the patients in group A was 5.23 ± 2.01 mol/L, that in group B was 6.14 ± 1.96 mol/L, and that in group C patients was 6.64 ± 1.87 mol/L. Obviously, the TC levels of patients in groups A and B were significantly different (*P* < 0.05). The TC levels of patients in groups A and C were also significantly different (*P* < 0.05), but the TC levels of patients in groups B and C were not significantly different (*P* > 0.05). After treatment, the average TC of patients in group A was 5.82 ± 1.47 mol/L, that in group B patients was 6.79 ± 1.77 mol/L, and that in group C patients was 5.33 ± 2.17 mol/L. There was no significant difference in TC values of patients in groups A and B before and after treatment (*P* > 0.05), while the difference in TC values of patients in group C before and after treatment was significant (*P* < 0.05).

### 3.6. Comparison of the MOCA Scores of the Three Groups of Patients before and after Treatment

**Figure 10:**
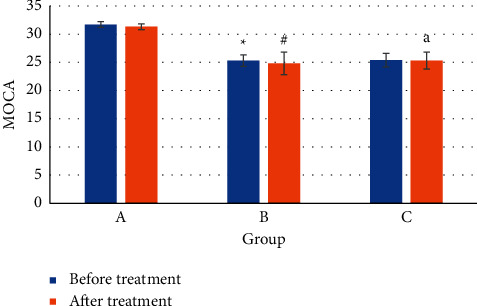
Comparison of MOAC levels before and after treatment in the three groups of patients. Note:  ^*∗*^ means there was a significant difference versus group A (*P* < 0.05). # indicates that there was a significant difference before and after treatment in the same group (*P* < 0.05). *a* means there was a significant difference versus group B (*P* < 0.05).

Figure 10 shows the MOCA scores of the patients before and after the treatment. Before treatment, the average MOCA of patients in group A was 31.7 ± 0.58, that in group B was 25.3 ± 1.96, and that in group C was 25.4 ± 1.02. Obviously, the MOCA scores of patients in groups A and B were significantly different (*P* < 0.05), and the differences in MOCA scores of patients in groups A and C were also statistically significant (*P* < 0.05), but the differences in MOCA scores between groups B and C were not significant (*P* > 0.05). After treatment, the average MOCA of patients in group A was 31.3 ± 0.79, that in group B was 24.8 ± 1.23, and that in group C was 25.3 ± 1.22. The differences in MOCA scores of group A were not statistically significant before and after treatment (*P* > 0.05). The MOCA scores in group B differed significantly before and after treatment (*P* < 0.05), and the difference in MOCA scores in group C was not significant before and after treatment (*P* > 0.05). After treatment, there was a significant difference in the MOCA scores between groups B and C (*P* < 0.05).

## 4. Discussion

Stroke is a cerebrovascular disease with high morbidity, disability, and mortality. Studies have shown that the prevalence of PSCI in patients with severe stroke is as high as 37%–80%, and it can be fatal in severe cases [15]. MRI is widely used in the examination and diagnosis of cerebrovascular diseases and has obvious advantages. However, in actual practice, it will be affected by objective factors such as the patient's breathing rate and the doctor's operations. The final MRI image quality is poor, so it is necessary to choose a suitable image segmentation algorithm [16]. In this study, the denoising degree of MRI images processed by the GMM-based LRMD algorithm was significantly improved, the image quality was significantly enhanced, and the diagnostic accuracy and efficiency of PSCI patients were heightened.

The increase in plasma HCY was related to cognitive impairment. Before treatment, the HCY levels of patients in groups A and B were significantly different (*P* < 0.05), and the HCY levels in groups A and C also differed significantly (*P* < 0.05), but there was no significant difference in HCY levels between groups B and C (*P* > 0.05). After treatment, the HCY values of patients in groups A and B were not significantly different before and after treatment (*P* > 0.05), and the HCY values of patients in group C were significantly different before and after treatment (*P* < 0.05). These results indicated that the HCY level of PSCI patients was significantly higher than that of non-PSCI patients, which was in line with the research conclusion of Ma et al. [17]. Folic acid is an important cofactor in the body's metabolic process. It can be supplemented with folic acid to reduce the level of HCY in the body and alleviate PSCI in patients [18]. After PSCI patients were treated with folic acid combined with atorvastatin, their own HCY levels were significantly reduced, which showed that folic acid combined with atorvastatin can lower patients' plasma levels, echoing the conclusions of previous studies.

Studies have shown that an increase in serum TC can inhibit brain metabolism by reducing cerebral blood flow, thereby increasing the probability of PSCI and dementia. In addition, high-level TC can also inhibit the deformation of related neurons to cause PSCI [19]. This is because the increase in serum TC will inhibit the metabolism of amyloid in nerve cells [20], resulting in the accumulation of amyloid protein in the patient's body, thus leading to PSCI. Meyer et al. [21] found that hyperlipidemia was a key cause of PSCI. In this study, it was found that before treatment, the TC levels of patients in groups A and B were significantly different (*P* < 0.05), and the differences in TC levels of patients in groups A and C were also significant (*P* < 0.05), but there was no significant difference in TC levels between groups B and C (*P* > 0.05). After treatment, the TC values of patients in groups A and B were not significantly different before and after treatment (*P* > 0.05), but the TC values of patients in group C were significantly different before and after treatment (*P* < 0.05). These results indicated that the increase in serum TC contributed to PSCI. The TC level of group C treated with folic acid combined with atorvastatin was significantly reduced. Therefore, folic acid combined with atorvastatin can reduce the plasma level of PSCI patients, thereby reducing PSCI.

The cognitive MOCA scores of the three groups of patients before and after treatment showed that after treatment, there was a significant difference in MOCA scores between the PSCI group treated by the folic acid combined with atorvastatin and the placebo-treated group B (*P* < 0.05). The scores of patients in group C decreased slowly, so the degree of PSCI in group C was smaller compared to group B. It is suggested that folic acid combined with atorvastatin can alleviate the PSCI of patients and can be used in the treatment of PSCI of stroke patients.

## 5. Conclusion

In this study, the GMM-based LRMD algorithm was applied to process MRI images of stroke patients. The images before and after processing were then compared. Next, the effects of atorvastatin combined with folic acid on the PSCI of patients with ischemic stroke were discussed in order to further understand the role of atorvastatin combined with folic acid in the PSCI and treatment of stroke patients. The results showed that the denoising degree of MRI images processed by the GMM-based LRMD algorithm was significantly improved, and the image quality was significantly enhanced. Atorvastatin combined with folic acid can reduce HCY, serum TC, and MOCA scores. Therefore, the processed MRI images have good quality, and folic acid combined with atorvastatin can effectively reduce plasma and serum levels, thereby reducing the PSCI of stroke patients. It was of great significance for the early diagnosis and treatment of stroke patients. However, some limitations should be noted. The sample size is small, which will reduce the power of the study, and an expanded sample size is necessary in the follow-up to strengthen the findings of the study [22].

## Data Availability

The data used to support the findings of this study are available from the corresponding author upon request.
